# Rheumatism

**Published:** 1845-04

**Authors:** 


### SELECTIONS

FROM

### AMERICAN AND FOREIGN JOURNALS.

---

*Rheumatism.*—It is startling, on the retrospect of a large hospital practice, to observe how many hard-working men and women perish miserably before their prime, from disorders of the pulse and breath, consequent on attacks of acute rheumatism. Of British fevers, with the exception of small-pox and scarlet fever, there is, I believe, not one of which, in the long run, and in a given number of cases, the result is more frequently fatal.

To all who are held professionally responsible for the safe conduct of this disorder to its close, the question must continually recur, whether such large contingent mortality may not, in part, be attributed to erroneous rules of practice. Assuredly, the analogy of definite and consecutive action, which we have sought to establish between the rheumatic and other forms of fever, is seldom regarded in its treatment. Under the "alias" of acute rheumatism, this disorder is subjected, in a twofold capacity, to direct interference from the practitioner during the entire process of its self-evolution. The patient is made to pay by the lancet for its acuteness, and swallows every specific for gout and neuralgia, in right of his rheumatism. Yet, in the approved treatment of rheumatic fever, though the character of its action be special and inflammatory, the principle of its cure is not antiphlogistic, or one of specifics. Obtained, as in the other continued fevers, from the observation of symptoms in their context and progression, it assists in the development of action, and is content, for the most part, to follow indications. In ordinary cases, it has been said that the fever of acute rheumatism is competent to the task of its own cure; yet no illness requires from the physician more careful and constant attention during its progress, for it is liable to many accidents and complications, with a sequel so harassing and protracted, that, of the patient, it is never known when he is fairly out of it. Moreover, it admits of great relief from various remedies, and may often be conducted, by judicious treatment, some days sooner, to its end; and when this is safe, it should always be done, for the case is never without danger while the fever lasts. Besides, with the means of relief in our hands, we cannot refuse, in this disorder, to prescribe for the pain—a symptom constantly present, and of proverbial severity. Here. in the administration of narcotic medicines, the principle of direct interference is at once admitted. From the indulgence of a false humanity, in getting rid of pain, without reference to its cause or context, and under the prevailing dread of all action, (in whatever kind or degree,) that is called by the bad name of "inflammation," there is reason to believe that many of the dangerous complications so frequent of late years in the pathology of acute rheumatism, do in truth proceed. In the well-known combination of calomel with opium, this mischievous diligence of treatment receives its most frequent illustration. The objects proposed in this heroic formula (when any are really entertained, for it began by caprice, and followed in the vogue) are not less than the immediate and complete extinction of fever, pain, and inflammation, with the consequent prevention of all dangerous complications, by organic lesion of the heart and other vital viscera. It is a rude empirical practice, which seldom succeeds, and, failing of success, is most injurious to the patient. In its routine application to the languid, weak, and exhausted, as to the full and vigorous habit of body, there is reason to believe that it has destroyed very many who, under less popular and energetic methods of treatment, would in due time have recovered.

It does not necessarily counteract inflammation of the heart, lungs, or pericardium. Again and again it has been observed in practice, that "heart symptoms" have been rapidly developed in cases of rheumatic fever, while under treatment by large and frequent doses of calomel in combination with opium. In many of these cases, is it not more than probable that the fevered masses of blood in circulation through the chest are directly influenced to their further prejudice by admixture with the drug and the mineral? With a full belief in the special curative agencies of mercury, yet knowing, in certain constitutions, how entirely it disagrees, and how dangerous it occasionally becomes from coincident effects of functional disorder or organic disease, we cannot be too careful in opposing it specifically to the fever of acute rheumatism. Calomel is not in any special relation of cure or relief with the rheumatic diathesis, and should not be employed in the simple rheumatic more frequently than in the simple continued fever. When I first entered on hospital practice, though continually deprecating the reckless use of mercury which then prevailed, I fell much into the habit of prescribing two grains and a half of calomel, with the fourth or the half of a grain of opium, at intervals of four or six hours, in the early stages of rheumatic fever. I have now for many years past discontinued the use of this compound alterative, and see no reason to recur to it.

There has been, of late years, such a crying up, among certain sectarians in London physic, of calomel in combination with opium, as specific in all inflammations, general, local, and special, that, holding this prescription on a threefold trust, we are compelled to it, on every count, in the cardiac aggravations of rheumatic fever.

Yet who could, with confidence, affirm that, by the employment of this formula, he had, in any one instance, prevented adhesion of the pericardium, or arrested the vegetations, by fibrinous deposit, on the cardiac auricular valves. Assuredly, under the free use of calomel with opium, the symptoms, as obtained by sound or otherwise, that are observed in coincidence with these states of the heart and its membranes, have not unfrequently been known to subside; but it is equally certain that the same effects of relief have followed, on very different and less energetic modes of treatment. The question remains, whether the mercurial salt, not being always needful in cardiac rheumatism, may not sometimes be injurious.

*London Lancet.*

